# Sexual Satisfaction among Married Women in Tiro Afeta District: A Cross-sectional Study

**DOI:** 10.4314/ejhs.v33i4.12

**Published:** 2023-07

**Authors:** Tigist Abebe Gelashe, Aderajew Nigusse Teklehaymanot, Bekelu Teka Worku

**Affiliations:** 1 Dimtu Primary Hospital, Tiru Afeta District, Ethiopia; 2 Jimma University, Department of Population and Family Health, Ethiopia

**Keywords:** Sexual, sexual satisfaction, married women, mixed method

## Abstract

**Background:**

Sexual satisfaction is directly related to marital sustainability and quality of life. This study assessed the magnitude of sexual satisfaction and associated factors among married women.

**Methods:**

A community-based cross-sectional study was conducted from May 18 to June 8/2021 using mixed data collection methods. The sample was calculated using single population proportion formula for the quantitative part while data saturation was applied for the qualitative part. Simple random and purposive sampling techniques were used to get participants for the quantitative and the qualitative parts respectively. The quantitative data were analyzed using Statistical Package for Social Science (SPSS) version 25, and the qualitative data were analyzed manually. Ordinal logistic regression was applied to explore the model. P-value <0.05 and AOR with a 95%CI were used to identify the statistical significance of the association.

**Result:**

About 398 married women participated in the study, making a response rate of 94.3% and 44.7% of them were moderately satisfied with their sexual life. Sexual satisfaction among the married women was significantly associated negatively with attending elementary education 99.9% [AOR=0.1, 95%CI:0.0,0.4)], positively with having social responsibility 19[AOR=19.3, 95%CI: 1.8, 28.3], and negatively with having poor sexual attitude 97%[AOR=0.1, 95%CI: 0.0, 0.3]. The qualitative finding showed that the majority of women engage in sexual intercourse for the satisfaction of their partners, without their needs.

**Conclusion:**

Sexual satisfaction among married women was low in the study area. Comprehensive sexual and reproductive health awareness and strengthening of the prevention of harmful traditional practices are recommended.

## Introduction

Sexual satisfaction is an individual or personal assessment of the level to which an individual is satisfied with his/her sexual activity (1). It is an emotional response arising from one's subjective evaluation of the positive and negative dimensions associated with one's sexual relationship (2).

Sexual satisfaction is an indicator used to measure sexual health. It is also directly related to marital sustainability and quality of life (3, 4). On the other hand, sexual health necessitates a constructive and humble technique to sexuality, sexual relationship, and the likelihood of having enjoyable and safe sexual practices. Furthermore, to achieve and sustain sexual health, the sexual rights of individuals must be respected, protected, and pleased. It also requires to be practiced and articulated in feelings, imaginations, wants, beliefs, attitudes, values, performances, roles, and interactions (4).

Sexual satisfaction is important for its various indispensable roles in human life. It is related to emotional satisfaction, mental health and physical health status, general happiness, overall life achievement, prevention of unsafe sexual activities, and good social relationships (5,6). In addition, sexual satisfaction increases the productivity of the women and sustains a marital relationship (7).

On the other hand, evidence shows that sexual satisfaction is the second most challenging issue among a sample of young married couples. Moreover, sexual dissatisfaction is a more frequent problem for women than men. Studies indicated that 15% to 50% of women in the world are not sexually satisfied (8,9). Similarly, sexual health concerns that are most commonly reported by women were related to their level of sexual desire (10). Evidences from different countries show that there is low sexual satisfaction among married women, in general (8,11-13). In Ethiopia, only half (50.4%) of married women enrolled in one study had sexual satisfaction (14).

In general, the literature suggests that sexual health should be managed, not just in the context of the human's age, gender, and general health, but also within their existing sexual relationship (10). Globally, attempts are made to improve sexual health by designing different strategies and programs. For example, one of the focus areas of sustainable development goal is ensuring the sexuality, sexual, and reproductive health needs of all people through preparing access to professional counseling and treatment for people with sexual dysfunction (15-17). Ethiopia also ratified those strategies and programs and developed guidelines on sexual and reproductive health (18). Despite that, sexual satisfaction and health remained today's women's problems. On the other hand, published articles are scarce on this issue in the study area as well as in Ethiopia among married women. The available studies focused on urban areas, quantitative approach only, and consequences of sexual dissatisfaction without addressing its prevalence. Consequently, this study aimed to assess sexual satisfaction and associated factors among married women in Tiru Afeta District, Ethiopia.

## Methods and Subjects

**Study area and period**: The study was carried out in Tiro Afeta District located in the Jimma Zone, Oromia regional state, Southwest Ethiopia, 284 kilometer from Addis Ababa, the capital city of Ethiopia. According to the 2021 data from the woreda health office, the total population of the District is estimated to be 170,641 (87,027 females and 83,614 males). About 41,283 of the female population are estimated to be married women. All available health facilities: one primary hospital and five health centers in the District were providing the packages of reproductive health services. Data collection was carried out from May 18/2021 to June 8/2021.

**Study design and population**: A community-based cross-sectional study using a quantitative data collection method supplemented with a qualitative method was employed.

**Sample size determination and sampling technique for the quantitative part**: The sample size was determined using single population proportion formula with the assumption of a 95% confidence level, 5% degree of precision, 50.4% proportion of women's sexual satisfaction in Kewot District (14), and 10% non-response rate. Finally, a sample size of 422 was used. The sample was proportionally allocated to the selected Kebeles depending on the number of married women available in the Kebeles. Participants of the study were selected using a simple random sampling method. Updated family folders of the Kebeles were used as a sample frame to apply simple random sampling.

**Sample size determination and sampling technique for the qualitative part**: Participants were selected by purposive sampling techniques for an in-depth interview. The selection was done with the guide of health extension workers and women's affairs in the Kebele based on the selection criteria of having a role in community work, the ability to express their idea, and those who feel free to express their ideas. Twenty married women who were not included in the quantitative part were included in the qualitative part based on the information saturation criterion

**Data collection tools and procedures for the quantitative part**: A structured questionnaire which was adapted from the “new sexual satisfaction scale (NSSS)” was used to collect data (19) for the magnitude of sexual satisfaction. NSSS is a useful tool for assessing sexual satisfaction regardless of a person's gender, sexual orientation, and relationship status (20). The measurement has seven major parts and sub-parts under each part. The new sexual satisfaction scale short form (NSSS-S) (K=12) has twelve questions on a 5-point scale with the answer for each question ranging from 1-not at all satisfied to 5-extremely satisfied (21). The questionnaire also includes questions related to factors associated with the sexual satisfaction of married women such as individual characteristics, and partners, marriage, social, and cultural related factors, and type of contraceptive used related factors adapted from relevant literature (8,19,22-28). All tools were prepared in English originally and translated into local languages Afan Oromo and Amharic by language experts. It was pretested on 10% of the sample in adjacent Kebele, and necessary modifications and corrections were made before the main data collection. Quantitative data were collected through a face-to-face interviewer-administered method by trained four female midwives. The data collection process was supervised by the other two female supervisors.

**Data collection guide and procedures for the qualitative part**: An interview guide with probing questions was prepared by the research team to collect data through an in-depth interview method. Place of an in-depth interview was prepared in respondents' living areas that can afford a maximum degree of confidentiality and privacy to them.

**Quantitative data processing and analysis**: Filled questionnaires were checked for completeness and missed information. Data were entered into Epi data version 3.1 and exported to SPSS version 25 for analysis. Further cleaning was done by running frequencies for each variable to identify and manage outliers and missing values. Descriptive statistics such as frequencies, percentages, means, and standard deviations were used to describe the findings and characteristics of the participants. To identify statically significant factors, univariable and multivariable ordinal logistic regression was done. Univariable ordinal logistic regression was used to identify factors associated with the dependent variable and fulfill the assumption of ordinal logistic regression analysis. Model Fitting Information (p-value <0.05), Goodness-of-Fit (Pearson p-value >0.05), Pseudo R-Square (Nagelkerke value), Parameter Estimates, and Test of parallel lines (p-value >0.05) were done. A multicollinearity check was done with a variance inflation factor (VIF) and was found to be 4.291. Factor analysis was conducted for the wealth status of the women where a total of 15 items were analyzed using principal component analysis methods. The fulfillment of the assumption for principal component analysis was assessed by Kaiser-Meyer-Olkin (KMO) measure of sampling adequacy =0.698 (>0.5), case to variables ratio 26 to 1 (above the standard 5 to 1), and significance of Bartlett's Test of Sphericity at P-value <0.00: there was a correlation. No variable had >=0.9 with an anti-image correlation matrix indicating there was no multicollinearity. In each step, variables with commonalities less than 0.5 and having a loading (>=0.4) in more than one component (having complex structure) were removed. The total variance explained by four components was 67.4%. A factor score of these components was used to classify women's wealth status into four groups. Reliability analysis was conducted and had a Cronbach's alpha of 0.779.

Finally, to identify the association between the independent and the dependent variables, all independent variables that fulfilled the assumption of the ordinal logistic regression model and were identified as the predictor variables in univariable ordinal regression at p-value < 0.25 were entered into the multivariable ordinal logistic regression model. The overall model to predict the level of sexual satisfaction of married women was statically significant at (p-value<0.05), and the overall prediction of the model was 70.8%. In multivariable ordinal logistic regression analysis, the presence of a statically significant association between the predictors and the outcome variables was declared at p-value <0.05 and AOR with 95% CI.

**Qualitative data processing and analysis**: Qualitative data were analyzed manually by using the thematic analysis method to triangulate the result with the quantitative result. Analysis was started by transcribing notes and audio records and translating them into English by the research team. The interpretation was drawn by triangulating both quantitative and qualitative results.

**Ethical approval**: Ethical clearance was obtained from Jimma University, Institute of Health Institutional Review Board with the number of IRB PG/101/2/2021. Study participants were informed of the objectives of the study and its method, and verbal consent was taken. For the participants who were less than eighteen years old, consent was obtained from themselves and the ascent was obtained from their partner. All of the participants' partners were greater than eighteen years old. They were informed that they have the full right to discontinue or refuse to participate at all. Data collection was anonymous, and information was preserved confidentially.

## Results

**Socio-demographic and economic characteristics of respondents**: A total of 398 married women participated in the study making a response rate of 94.3%. The majority of the participants were in the age group of 26-35 years. Less than one-third (28.6%) of the respondents were in the first quintile of the wealth index (Table 1).

**Table 1:** Distribution of socio-demographic characteristics of study participants (n=398)

Variable	Category	Frequency	Percent
	15-25	101	25.3
Age category	26-35	255	64.1
	>=36	42	10.6
Educational status of respondents	Unable to read and write	204	51.3
	Able to read and write	105	26.3
	Elementary school	59	14.8
	High school	7	1.8
	Diploma and above	23	5.8
	Unable to read and write	203	51
Educational status of husbands	Able to read and write	91	22.9
	Elementary school	70	17.6
	High school	6	1.5
	Diploma and above	28	7.0
	Housewife	339	85.2
Occupational status of respondents	Merchant	18	4.5
	Daily labourer	5	1.2
	Government employee	36	9.1
	First quintile	114	28.6
Wealth status of respondents	Second quintile	84	21.1
	Third quintile	98	24.6
	Fourth quintile	102	25.6
Number of children	I have no child	33	8
	only one	87	22
	2-5	217	55
	6-10	57	14
	>=10	4	1
Duration of marriage	1-10 years	274	68.8
	11-15 years	62	15.6
	>= 15 years	62	15.6
Frequency of sexual intercourse in the last month	Never	8	2
	1-4 days	168	42.2
	5-15 days	155	39
	15-25 days	67	16.8

**Figure 1:**
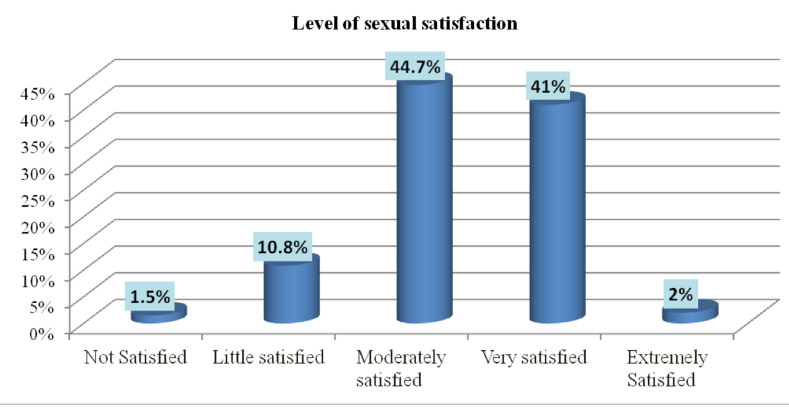
Distribution of level of sexual satisfaction among study participants

**Prevalence of sexual satisfaction**: Among the 398 women who participated in this study, 178(44.7%) and 163(41%) were moderately and very satisfied with their sexual life respectively. On the other hand, about 1.5% of the women were not satisfied at all with their sexual life (Figure 1).

**Figure 2:**
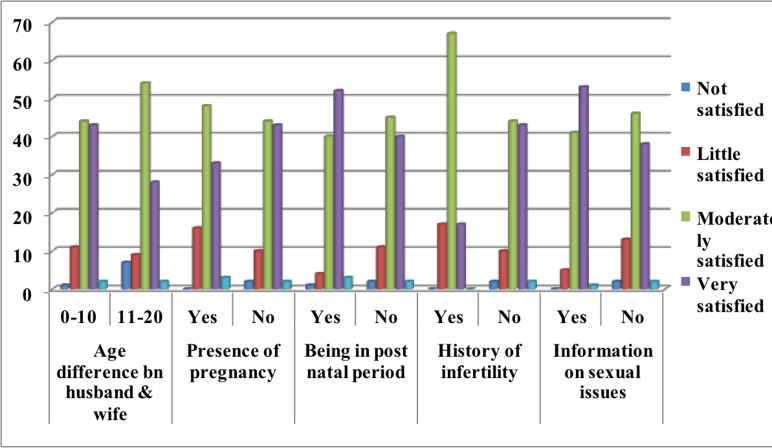
Individual characteristics of married women who participated in the study

**Individual characteristics of respondents**: About 352 respondents had 0-10 years of age difference from their husbands. Around two percent of the participants had identified infertility problems. Sixteen respondents (4%) chewed khat at least once a day. More than half of the respondents had poor sexual attitudes and sexual self-esteem (Figure 2).

In the qualitative finding of this study, most of the study participants reported that individual characteristics of the women and their partners can affect women's sexual satisfaction. Many of them said the age difference between husband and wife should not be more than 10 years. As the age difference became more, understanding each other can be more difficult, and they fail to agree on ideas. In addition to this, women are afraid to discuss their feelings and emotions with their husbands.

“*My husband is approximately fifteen years older than me. I married him under my family pressure. I never discuss sexual issues with him; we do have sexual intercourse only at a time he wants. I do not think he remembers me while he is enjoying himself. He does not worry about my desire. He expresses his interest only. However, I never blamed him for this issue yet because I have simply accepted this as our culture though I do not have pleasure from having sexual intercourse with him at all*.” (Participant 5)

“*I am about five years younger than my husband. Both of us had attended secondary school. We always discuss different issues which are important to our life without any worry. We love each other, and there is nothing hidden between us. I tell him what I want and he also does. We are happy with all aspects of our life including sexual life*.” (Participant 7)

**Social and partner relationship and contraceptive use characteristics of study participants**: About two-thirds (66.3%) and one-fourth (26.9%) of the respondents had good and very good social relationships in their community respectively. Among those who had good and very good relationships, 41% and 47% of them were very satisfied with their sexual life respectively. Additionally, 17.3% of the respondents had a social role in the community, and more than half of them were very satisfied with their sexual life (Table 2). The qualitative finding in this study is in line with this result where the majority of the participants raised ideas that support social roles to have a positive connection with sexual satisfaction. They described that social responsibility can help women to be active in all aspects of their life. As the woman participates in different social roles, she develops self-confidence, gets exposure to different types of information, boosts her ability to manage inside and outside of home responsibilities in a better way and become capacitated on the ways of expressing her idea and emotions.

**Table 2:** Social relationship, partner relationship and contraceptive use characteristics and level of sexual satisfaction of married women

Variables	Categories	Not satisfied	Little satisfied	Moderately satisfied	Very satisfied	Extremely satisfied
**Social relationship**	Bad	0	0	1(50)	1(50)	**0**
	Neither bad nor good	2 (8)	7(28)	13(52)	3(12)	**0**
	Good	3(1)	16(6)	133(50)	109(41)	**3(1)**
	Very good	1(1)	20(18.6)	31(29)	50(47)	**5(4.6)**
**Social role**	Yes	1(1.4)	1(1.4)	29(42)	36(52.2)	**2(3)**
	No	5(1)	42(13)	149(45)	127(39)	**6(2)**
**Being effective in social role**	Yes	1(2.2)	0	13(28.3)	30(65.2)	**2(4.3)**
	No	0	1(4)	16(70)	6(26)	**0**
**Interpersonal communication with partner**	Poor	6 (4)	31 (19)	100 (62)	23 (14)	**1 (1)**
	Good	0	12 (5)	78 (33)	140 (59)	**7 (3)**
**Sexual attachment with a partner**	Poor	5 (3)	27 (16)	87 (51)	47 (28)	**3 (2)**
	Good	1(0.4)	16 (7)	91 (40)	116 (51)	**5 (2)**
**Sexual Function with a partner**	Poor	6 (3)	32 (17)	98 (54)	45 (25)	**2 (1)**
	Good	0	11 (5)	80 (37)	118 (55)	**6 (3)**
**Sexual self-confidence with partner**	Poor	6 (3)	35 (19)	108 (58)	36 (19)	**2 (1)**
	Good	0	8 (4)	70 (33)	127 (60)	**6 (3)**
**Sexual attitude with partner**	Poor	6 (3)	35 (17)	109 (52)	56 (27)	**3 (1)**
	Good	0	8 (4)	69 (36.5)	107 (56.6)	**5 (3)**
**Sexual self-esteem with partner**	Poor	6 (3)	35 (17)	107 (53)	50 (25)	**3 (2)**
	Good	0	8 (4)	71 (36)	113 (57)	**5 (3)**
**Use of contraceptive**	Yes	4(2)	16(6)	116(46)	112(44)	**4(2)**
	**No**	**2(1)**	**27(18)**	**62(42)**	**51(35)**	**4(3)**

“I *can talk to my husband as he talks to me. As I have got experience in different training sessions, I believe I differ from my husband only with my sex. I have a social responsibility in this Kebele. I am trained in leadership as early as I took the responsibility. I have participated in different meetings and training that help me to learn many things from different people. Hence, I am not afraid to talk to my husband who is more relevant to me than anyone else. I am sure this helped me to have a satisfying sexual life.”* (Participant 11) *“I have social responsibility. This work makes me too busy. Consequently, most of the time, I share my relatively free time which is in bed, to enjoy love with my husband. At that time, we do whatever we want to do. Even if it makes me busy, I learn a lot from this responsibility which helps me in my life. I do not want to criticize my husband at every moment. If there is a problem, we discuss it and solve it smoothly. He understands my interest and tries to make me happy. Hence, I am happy with my sexual life.”* (Participant 12)

More than half (60%) of the respondents had good interpersonal communication, sexual attachment, and sexual function whereas 59% of them were very satisfied with their sexual life. Moreover, the majority of the women who had at least one child reported that this interfered with their sexual life and satisfaction. According to the qualitative finding, many of the participants complained that having many children decreases sexual desire and satisfaction. This could be because as the number of children increases, responsibilities and in-house burdens come to the women. Subsequently, the woman can become exhausted due to many activities at home and taking care of her children. This in turn can decrease her sexual desire and satisfaction (Table 2).

“*I have 5 children. I am extremely busy throughout the day. I have many activities to do at home without any rest. At night, I want to sleep. I do not want to have sexual intercourse. Following this, my husband married another younger woman, and most of the time, he spent his time with her and slept there in the night also. There is no resting and recreation time I have. In general, for the last three years, I do not have sexual satisfaction*.” (Participant 1)

“*I am a mother of 7 children. I want to sleep with my children. My sexual desire is decreasing from time to time. I cannot tell you any tangible reason. Nevertheless, I do not want to have sexual intercourse. If I have intercourse, it must be on the need of my husband. I am not happy with that*.” (Participant 9)

Among the 63% of married women who used any contraceptive method, 46% were moderately satisfied with their sexual life (Table 2).

**Figure 3:**
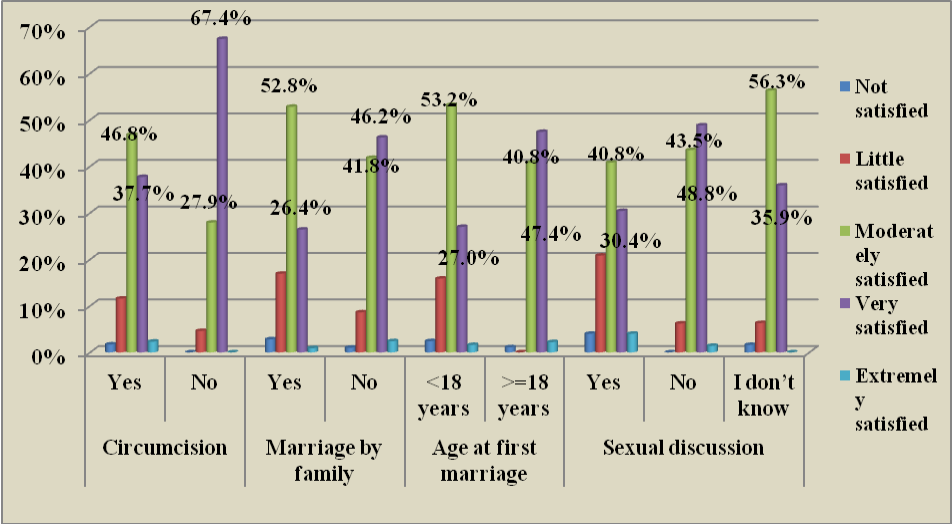
Cultural characteristics and the level of sexual satisfaction of married women

**Cultural characteristics of respondents**: More than one-third (32%) of the participants were married at less than 18 years of age, and only 27% of them were very satisfied with their sexual life. The majority of the respondents were circumcised among which 46.7% and 37, 7% were moderately and very satisfied respectively (Figure 3). In the qualitative finding, cultural practices such as female genital mutilation, marriage by family pressure, and early marriage were among the main issues raised repeatedly by participants as the reasons for decreasing sexual satisfaction among married women.

“*I was circumcised. Currently, we have two children with my husband. Nonetheless, I am not happy with my sexual life throughout this movement in marriage. I feel pain during sexual intercourse. I usually worry about my life rather than feeling happy during intercourse*.” (Participant 2)

*“I was married by family choice and their pressure. I didn't know my husband previously/before marriage. I have never been in love with him in my life. I cannot sense anything during intercourse. Because of this, I am not happy in my life at all, not only in my sexual life.”* (Participant 8)

“*I was married at 17 years of age. During sexual intercourse, I always feel severe pain. Usually at night, I cry and I have no sexual desire. I have told my problem to my husband several times. However, he ignored my words as he thinks that I am cheating him. After I gave birth to my first child, there is some improvement. Though, I am not satisfied with my sexual life*.” (Participant 6)

**Factors associated with sexual satisfaction**: According to the univariable ordinal logistic result, the age of respondents, educational status of respondents and their husbands, occupation, being in the postnatal period, having information on sexuality, being circumcised, early marriage, marriage by family pressure, age at first marriage, duration of the marriage, carrying out social responsibility, use of contraceptives, sexual self-esteem, sexual-attitude, sexual self-confidence, sexual function, sexual attachment and communication with a partner were identified as candidate variables. In multivariable ordinal logistic regression analysis, the educational status of the respondents, having social responsibility/role, and poor sexual attitude were significantly associated with the level of sexual satisfaction among married women in different directions of association.

Accordingly, the odds of moderate sexual satisfaction among married women who had attended elementary school education was 99.9% times [AOR=0.1, 95% CI:(0.0,0.4)] less likely compared to those who had a diploma and above educational level. On the other hand, the odds of moderate sexual satisfaction among married women who had social responsibility was 19 times [AOR=19.3, 95% CI :( 1.8, 28.3)] more likely compared to married women who had no social responsibility. Furthermore, the odds of extreme sexual satisfaction among married women who had poor sexual attitudes were 97% times [AOR=0.1, 95% CI: (0.0, 0.3)] less likely compared to those women who had good sexual attitudes (Table 3).

**Table 3:** multivariable ordinal logistic regression analysis result showing factors associated with the level of sexual satisfaction

Variable and Category	Level of sexual satisfaction					COR (95% CI)	AOR (95% CI)
	Not (%)	Little (%)	Moderate (%)	Very (%)	Extreme (%)		
**Educational status of respondents**							
Unable to read and write	5(2.5)	25(12.3)	100(49)	72(35.3)	2(0.9)	0.2(0.1, 0.4)	**0.1(0.0,7)**
Read and write	0	9(8.6)	50(47.6)	45(42.9)	1(0.9)	0.2(0.1, 0.5)	**0.01(0.0,6)**
Elementary	1(1.7)	7(11.9)	22(37.3)	25(42.4)	4(6.8)	0.3(0.1, 0.7)	0.1(0.0,0.4)^*^
High school	0	1(14.3)	3(42.9)	3(42.9)	0	0.2(0.1,1.0)	**0.2(0.1,1.0)**
≥Diploma	0	1(4.3)	3(13)	18(78.3)	1(4.3)	1	**1**
**Social responsibility**							
Yes	1(1.5)	1(1.5)	29(42)	36(52.2	2(2.9)	5.9 (2, 18.2)	19.3(1.8,28.3)^*^
No	5(1.5)	42(12.8)	149(45.3)	127(38.6)	6(1.8)	1	**1**
**Communication with partner**							
Poor	6(4)	31(19)	100(62)	23(14)	1(1)	0.12(0.1,0.2	**3.7(0.2,85)**
Good	0	12 (5)	78(33)	140(59)	7(3)	1	**1**
**Sexual attitude**							
Poor	6(3%)	35(17)	109(52)	56(27)	3(1)	0.26(0.2,0.4	0.1(0.0,0.3)^*^
Good	0	8(4)	69(37)	107(57)	5(2)	1	1

* Statically significance at p-value < 0.05

The qualitative finding shows that many of the participants said that discussing sexual issues with their husbands, self-confidence of women, and good sexual attitude is important for women to have sexual satisfaction. If the woman follows only her husband's interest, she is never satisfied with her sexual life.

“*I was married three years ago. I believe that having sexual intercourse can bring happiness to my life. It helps me to have a good relationship with my husband. I am satisfied with my sexual life because as I understand my husband's needs and feelings, he also understands mine*.” (Participant 30)

*“I know that sexual intercourse has advantages to my life. It is the way of getting a child. It helps to decrease stress and makes me happy. It increases the bond between me and my husband. Hence, it is very important for our futurity and the sustainability of our marriage”. Believing in this way, I am happy with my sexual life.”* (Participant 10)

## Discussion

This study identified the level of sexual satisfaction and its associated factors among married women in the Tiro-Afeta District. Sexual satisfaction is crucial for a strong and stable marital relationship as well as for the mental health of women (6,29,30). However, it can be affected by different factors such as the interaction of biotic, psychosomatic, communal, financial, political, traditional, moral, historical, spiritual, and mystical features and local factors which include cultural and social aspects of couples (28,31,32).

**Level of sexual satisfaction**: The magnitude of moderately sexually satisfied (44.7%, 95% CI: 43.2,45.5) married women in this study is relatively higher as compared with study findings conducted in Nigeria (42%) and Ghana (34%) (12, 33). These differences might be because of the difference in the tools used to assess the magnitude of sexual satisfaction. Moreover, differences in the year of study can also create discrepancies among the results as the ability of the women to talk about sexuality and report on related issues can be improved from time to time due to increasing accessibility of the information on the subject.

However, sexual satisfaction in this study is relatively lower than the study findings from Iran (57.8%), China (63%), Germany (57%), and Chile (66%) (13, 27, 34, 35). Such considerable differences might be a result of sociocultural differences and differences in accessing sexuality information since all of those studies were conducted in developed countries. Additionally, unlike this study, many of those studies were conducted in institutions and among educated participants which shows that there is variation in educational status among study participants. Moreover, data were collected by self-a administered method in those studies which is a better approach to increase the true report of such a sensitive issue by respondents. In the current study, there may be under-reporting from the presence of cultural sensitivity; it is taboo to express sexual feelings, especially by women in Ethiopia (36,37).

A study conducted among married women in Northern Ethiopia also reported higher (50.4%) sexual satisfaction than the finding of the current study. This lower result might relate to the socio-cultural difference between the study areas. In the current study area, khat chewing and polygamy are more common which is not common in the northern part of Ethiopia (38). Khat-chewing and polygamy are among the common factors which can reduce sexual desire and satisfaction by decreasing couples' relationships(39, 40).

**Factors associated with sexual satisfaction**: This study identified three variables that are statically significantly associated with the level of sexual satisfaction among married women. Accordingly, the educational level of the respondents was significantly associated with the level of sexual satisfaction. This finding is similar to a previous study conducted in Pakistan which explains that education equips a woman to deal with her sexuality in a better way leading to sexual satisfaction in a relationship. Education provides women with a chance of comprehending different aspects of their sexual and reproductive life thereby increasing their sexual satisfaction (4). Furthermore, studies conducted in Iran at different times showed that as the level of education increases, the rate of sexual pleasure also increases. It was also identified that women who attended university education expressed more sexual satisfaction than illiterate women (6,27). This finding can be due to the fact that, as the educational level of women increases, knowledge of sexuality can increase, sexual self-confidence grows, and sexual self-esteem is enhanced from better access to sexual information. Parallelly, the level of understanding of different aspects of reproductive and sexual health can increase (41). In addition, as the educational level of a woman increases, she can easily communicate with her partner and can have a good interpersonal relationship. In the qualitative study, most of the participants frequently explained that women's education has crucial role to have sexual satisfaction. According to the respondents, uneducated and less educated women do not speak out about their sexual needs and feelings to their partners.

“I had completed elementary school. I was married to a man whom I love by my interest. During sexual intercourse, I was feeling high pain. I went to my nearby health facility and they gave me some useful advice. I did what they told me. After that, the pain is decreased. Currently, I am having sexual pleasure.” (Participant 4)

Furthermore, respondents who had participated in social activities, a social role, and effective in social roles were more satisfied with their sexual life. Similarly, a study conducted in the United States of America found that having a satisfactory sexual life needs the skill of interpersonal communication, conflict resolution, and social interaction. These skills help the woman to develop self-esteem, self-confidence, and self-concept which can guide her to be happy in her sexual life (25). Sexual satisfaction and having social responsibility have a direct relationship because participating in social activities contribute to the improvement of women's lives in many aspects as they have exposure to different source which can help them to have good life skill. They can get an improved ability to manage challenges and better interpersonal communication skills which helps to discuss sexual issues at a different level and be sexually satisfied.

In this study, sexual attitude had a significant association with the sexual satisfaction of married women. Similar with this finding, in a study conducted in Iran, women with good attitudes toward sexual activity were inclined to experience more sexual satisfaction for the reason that a good sexual attitude can increase sexual behaviors, sexual frequency, and ability to deal with the sexual dysfunction of their partners (42). In the same way, a study conducted in the United States of America was in line with the current result showing good sexual attitude results in a better level of sexual pleasure, self-reliance in a sexual relationship, sexual ease, and sexual success that has a strong relation with sexual satisfaction (43). Sexual satisfaction and good sexual attitude can have an association because sexual satisfaction necessitates physical and mental readiness (44). Among the dimensions of mental preparedness, having a positive attitude toward sexuality is the most important one. Women who had very narrowly defined social roles (high satisfaction/high activity women) reported high satisfaction and high activity, as they can successfully fulfill these social roles.

In conclusion, less than half of married women were moderately satisfied whereas few were not satisfied at all with their sexual life. The qualitative finding showed that the majority of women engaged in sexual intercourse for the satisfaction of their partners even without their needs. Having children, more than ten years of the age difference between the couples, circumcision, early, and family-imposed marriage decrease sexual satisfaction while participation in community roles increases the sexual satisfaction of married women. It is recommended to consider sexual satisfaction as one of the public health importance. Also, the authors recommended sexual education intervention in the area particularly, on pre-marriage and within-marriage counseling.
